# The Efficacy of Renal Denervation in Treating Resistant Hypertension: A Systematic Review

**DOI:** 10.7759/cureus.67007

**Published:** 2024-08-16

**Authors:** Gibran A Azeez, Mounika Thirunagari, Nazeefa Fatima, Abhinav Anand, Aadi R Palvia, Avneet Kaur, Sondos T Nassar

**Affiliations:** 1 Department of Internal Medicine, California Institute of Behavioral Neurosciences and Psychology, Fairfield, USA; 2 Department of Pathophysiology, St. George's University, St. George's, GRD; 3 Department of Internal Medicine, Davao Medical School Foundation, Davao City, PHL; 4 Department of Clinical Research, California Institute of Behavioral Neurosciences and Psychology, Fairfield, USA; 5 Department of Internal Medicine, Kharghar Medicity Hospital, Navi Mumbai, IND; 6 Department of Medicine and Surgery, Jordan University of Science and Technology, Amman, JOR

**Keywords:** uncontrolled blood pressure, sympathetic denervation, systemic hypertension, renal ablation, treatment-resistant hypertension, renal denervation therapy

## Abstract

Resistant hypertension is blood pressure (BP) that is persistently above target in spite of the maximally tolerated usage of at least three anti-hypertensives simultaneously. The sympathetic nervous system is instrumental in blood pressure (BP) regulation. Renal (sympathetic) denervation involves using ablative energy to disrupt the sympathetic nerves in renal arteries. This systematic review examines the efficacy of this treatment modality. Abiding by the Preferred Reporting Items for Systematic Reviews and Meta-Analyses (PRISMA) 2020 guidelines, we conducted an extensive literature search in five databases, Cochrane Library, Google Scholar, PubMed, PubMed Central (PMC), and ScienceDirect, to retrieve studies that are free, open access, and published in English done within the past four years. Nineteen articles passed critical appraisal. These articles were randomized controlled trials (RCT), a case report, a cross-sectional study, a cohort study, and previous reviews. Renal denervation (RDN) was generally superior to sham control in patients with resistant hypertension for reducing various systolic blood pressure (SBP) measures, including 24-hour ambulatory, daytime, and nighttime SBP. The efficacy was highest in patients whose baseline SBP was higher. BP reduction was sustained for years post-procedure. The procedure had a good safety profile with no severe complications. Future studies should compare the efficacy of different types of renal denervation, such as ethanol ablation versus radiofrequency ablation, and renal denervation against other procedure-based treatment modalities, such as carotid baroreceptor stimulation and transcranial direct current stimulation.

## Introduction and background

According to the 2017 American College of Cardiology (ACC) and the American Heart Association (AHA) blood pressure (BP) treatment guidelines, hypertension is blood pressure that is 130/80 mmHg or higher [1]. The World Health Organization reports that approximately 1.28 billion people around the world have hypertension [2]. This demonstrates that hypertension remains a significant global health concern. There is an especially concerning type of hypertension: resistant hypertension. In a scientific statement from the American Heart Association, resistant hypertension is defined as blood pressure that is persistently elevated above target despite a patient's concurrent use of at least three anti-hypertensive agents, each of different classes, administered at maximally tolerated doses [3,4]. It should be noted that before diagnosing a patient with resistant hypertension, pseudo-resistant hypertension (pseudo-RH) should be excluded. Pseudo-RH has a variety of causes, including non-compliant adherence to anti-hypertensives and inadequate dosing of anti-hypertensives [5]. The underlying pathophysiology of hypertension and resistant hypertension is in part due to the imbalances in the renin-angiotensin-aldosterone system. Current anti-hypertensive guidelines have long featured drugs targeting this system, such as angiotensin-converting enzyme (ACE) inhibitors and mineralocorticoid receptor antagonists. However, there is another important link that underpins hypertension: the sympathetic nervous system [6].

The second key component of the pathophysiology of resistant hypertension is the activation of the sympathetic nervous system. Anti-hypertensives that act on the sympathetic nervous system are fifth-line agents in hypertension guidelines [7]. Current literature suggests that starting hypertension treatment with beta‐blockers provides a notable decrease in cardiovascular disease and negligible effects on mortality. Of note, beta‐blockers are inferior to those of other anti-hypertensive drugs [8]. Naturally, it is not surprising that beta-blockers are not first-line agents. Even so, the significant role of the sympathetic nervous system in hypertension has led to the development of innovative novel treatment modalities, termed device-based therapies. One such modality is renal sympathetic denervation. Renal sympathetic denervation refers to the ablation of renal afferent and efferent nerves through a catheter‐based percutaneous intervention performed via femoral access. The ablation method can be via low‐dose radiofrequency or focused ultrasound energy that disrupts the nerve fibers located at the walls of renal arteries [9].

Renal denervation (RDN) was a promising treatment modality when it was first under study, but follow-up trials rendered its status controversial and inconclusive. There were discrepancies between earlier and subsequent trials about the efficacy of renal denervation. Such discrepancies necessitated further clinical trials and ongoing systematic reviews of recent findings [10]. Given the small sample size and shorter follow-up period in earlier studies, it is promising and necessary that newer studies are being undertaken and longer follow-ups can be done [11]. New, recent studies provide an opportunity for a more comprehensive review and judgment on the efficacy of renal denervation. As an editorial comment in the Journal of the American College of Cardiology notes, interest in renal denervation is on the rise again [12], and we, the authors of this current systematic review, are certainly interested.

## Review

Methodology

This systematic review was undertaken in agreement with the outline of the Preferred Reporting Items for Systematic Reviews and Meta-Analyses (PRISMA) 2020 guidelines [13].

Eligibility Criteria

The studies were selected based on the Participants, Intervention, Control, and Outcomes (PICO) elements. Note that this study was a systematic review, and controls were optional. The population or participants were patients with resistant hypertension. The intervention was renal denervation. The outcome was any measure of blood pressure change.

Additional filters were applied as follows: inclusion criteria included studies published from 2021 to 2024, studies for which the free full text was available, and studies published in English. The choice of the years 2021 to 2024 reflects our aim to review more recent developments in the literature. Exclusion criteria were animal studies, studies in other languages, and studies older than the specified time. Additionally excluded were studies unrelated to any component of the PICO research question, such as participants being outside of the chosen population, or not receiving the intervention.

Databases and Search Strategy

We conducted our search systematically using PubMed, PubMed Central (PMC), Cochrane, Google Scholar, and ScienceDirect as the chosen databases. The last date of the search for all databases was May 4, 2024. The key terms employed in the five search engines were renal denervation and resistant hypertension. The field search used in the process was selected via Boolean keywords or Medical Subject Headings (MeSH), depending on the database, as summarized in Table 1. The table also displays the inclusion criteria.

**Table 1:** Databases and search strategy outlined MeSH: Medical Subject Headings, PMC: PubMed Central

Databases	Keywords	Search strategy	Number of articles before filters	Filters	Search result
PubMed	Renal denervation, resistant hypertension	Mixed MeSH: renal denervation OR renal sympathetic denervation OR renal ablation OR sympathetic denervation OR ("denervation/instrumentation"[Majr] OR "denervation/methods"[Majr]) OR ("sympathectomy/instrumentation"[Majr] OR "sympathectomy/methods"[Majr] OR "sympathectomy/statistics and numerical data"[Majr] OR "sympathectomy/trends"[Majr]) AND resistant hypertension OR refractory hypertension OR "hypertension resistant to conventional therapy" [Supplementary Concept] OR ("hypertension/epidemiology"[Majr] OR "hypertension/pathology"[Majr] OR "hypertension/physiopathology"[Majr] OR "hypertension/radiotherapy"[Majr] OR "hypertension/therapy"[Majr])	145,796	2020-2024, English language, free full text, human studies, all article types	1,770
Google Scholar	Renal denervation, resistant hypertension	Keyword Boolean: "renal denervation" OR "renal sympathetic denervation" OR "renal ablation" AND "resistant hypertension" OR "refractory hypertension" OR "hypertension resistant to conventional therapy"	9,020	Articles from 2021-2024, all study types	2,140
ScienceDirect	Renal denervation, resistant hypertension	Keyword Boolean: "renal denervation" OR "renal sympathetic denervation" OR "renal ablation" AND "resistant hypertension" OR "refractory hypertension" OR "hypertension resistant to conventional therapy"	5,846	Articles from 2021-2024, all study types, English, open access	384
Cochrane Library	Renal denervation, resistant hypertension	Keyword Boolean: renal denervation OR renal sympathetic denervation OR renal ablation AND resistant hypertension OR refractory hypertension OR hypertension resistant to conventional therapy	1,792 Cochrane trials, 13 Cochrane reviews	2021-2024, English	316 Cochrane trials, 5 Cochrane reviews
PMC	Renal denervation, resistant hypertension	Keyword Boolean: "renal denervation" OR "renal sympathetic denervation" OR "renal ablation" AND "resistant hypertension" OR "refractory hypertension" OR "hypertension resistant to conventional therapy"	4,121	2021-2024, open access	1,193
Total					5,808

Results

**Figure 1:**
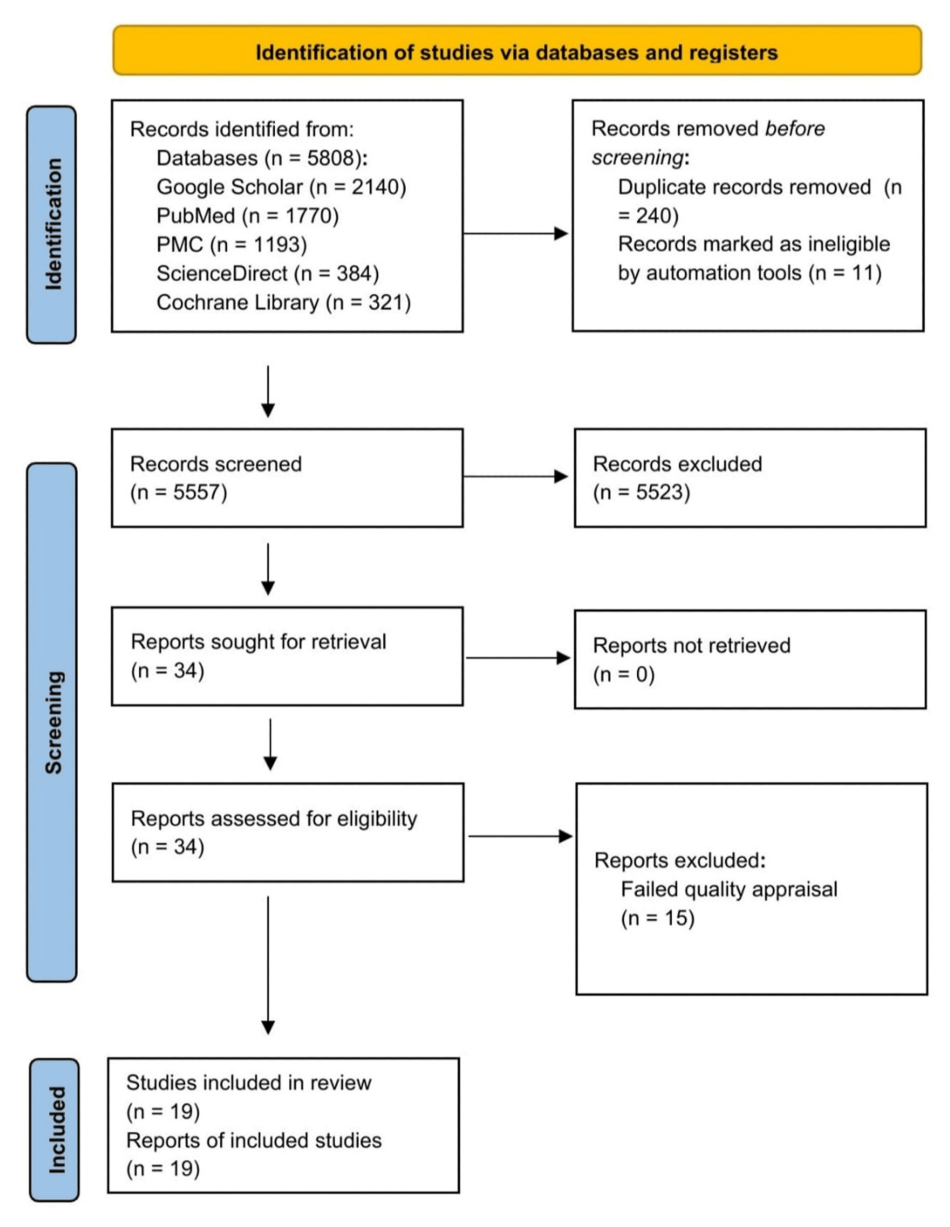
PRISMA 2020 flow diagram illustrating the screening process for this systematic review PRISMA: Preferred Reporting Items for Systematic Reviews and Meta-analyses [13]

From the database search, a grand total of 5,808 articles were compiled. All references were grouped and organized using EndNote 21 (Clarivate Analytics, Philadelphia, PA). A total of 240 duplicates were removed. Eleven articles were removed automatically by EndNote 21. Thereafter, the remaining 5,557 records were manually screened based on the titles and abstracts, excluding irrelevant studies. This stage removed 5,523 articles, leaving 34 studies to be retrieved as free full texts. After this review stage, the 34 full-text articles were retrieved for the next stage: quality appraisal. After completion of the quality appraisal, only 19 studies were included in the final review. Figure 1 displays the overview of this process.

Quality Appraisal

The full texts retrieved were critically appraised for methodological quality and any risk of bias using the appropriate tool per study type. Two reviewers independently appraised the articles, and where discrepancies occurred, a third reviewer was consulted. The tools used were as follows. For literature reviews, the Scale for the Assessment of Narrative Review Articles (SANRA) was chosen [14]. All systematic reviews utilized the Assessing the Methodological Quality of Systematic Reviews (AMSTAR 2) [15]. The cross-sectional study utilized the Appraisal tool for Cross-Sectional Studies (AXIS) [16]. The case report was assessed using the Joanna Briggs Institute (JBI) Critical Appraisal Checklist for Case Reports [17]. All cohort studies used the Newcastle-Ottawa Scale (NOS) for Cohort studies [18]. The randomized controlled trials (RCTs) were assessed using the Revised Tool to Assess Risk of Bias in Randomized Trials (RoB 2) [19]. The remaining studies utilized either the Quality Assessment Tool for Before-After (Pre-Post) Studies With No Control Group [20] or the Risk of Bias in Non-randomized Studies - of Interventions (ROBINS-I) [21].

For the eight narrative/literature reviews eligible for quality appraisal, the Scale for the Assessment of Narrative Review Articles (SANRA) tool was used. This six-item tool had possible scores of 0, 1, or 2 for each item and a maximum score of 12. Only studies that scored at least 9/12 (>70%) were accepted. As shown in Table 2, seven narrative reviews passed the critical appraisal.

**Table 2:** Summary of SANRA SANRA: Scale for the Assessment of Narrative Review Articles [14]

First author, year	Study importance justified	Concrete aims/questions	Literature search described	Referencing	Scientific reasoning	Appropriate data presentation	Appraisal
Champaneria et al. (2023) [22]	2	2	2	2	2	2	12/12
Dybiec et al. (2023) [23]	2	2	1	2	2	1	10/12
Guber et al. (2022) [24]	2	2	1	2	2	2	11/12
Liang et al. (2021) [25]	2	2	2	2	2	1	11/12
Pan et al. (2021) [26]	2	2	1	2	2	1	10/12
Schmieder et al. (2023) [27]	2	0	1	2	2	2	9/12
Persu et al. (2023) [28]	2	2	2	2	2	2	12/12
Xiong et al. (2023) [29]	0	1	2	2	2	1	8/12

Six systematic reviews and meta-analyses were checked using the Assessing the Methodological Quality of Systematic Reviews (AMSTAR). To be deemed of sufficient quality, a study required a minimum of 12 points, which is greater than 70% of the total score (one "yes" = 1 point, a "partial yes" = 0.5 points). As shown in Table 3, five studies passed and one study failed. The failed study did not address key items of AMSTAR such as adequately accounting for risk of bias or explaining any heterogeneity in the results.

**Table 3:** Summary of AMSTAR AMSTAR: Assessing the Methodological Quality of Systematic Reviews [15] PICO: Participants, Intervention, Control, and Outcomes, Y: yes, N: no, PY: partial yes

Checklist	Ahmad et al. (2021) [30]	Li et al. (2023) [31]	Pisano et al. (2021) [9]	Sharp et al. (2024) [32]	Silverwatch et al. (2021) [33]	Singh et al. (2022) [34]
Inclusion of PICO	Y	Y	Y	Y	Y	Y
Methods established prior to review	Y	N	N	Y	Y	Y
Explanation of study design chosen	Y	N	N	Y	N	N
Literature search strategy	Y	Y	Y	Y	Y	Y
Duplicate study selection	Y	Y	Y	Y	Y	Y
Duplicate data extraction	Y	Y	Y	Y	Y	Y
Justified list of excluded studies	N	N	Y	N	N	N
Description of included studies	Y	Y	Y	Y	Y	PY
Risk of bias assessment	Y	Y	Y	Y	Y	N
Funding for studies	N	N	Y	N	N	N
Appropriate statistical result combination	Y	Y	Y	Y	Y	N
Impact of bias assessed	Y	Y	Y	Y	Y	N
Accounting for risk of bias	Y	Y	Y	Y	Y	N
Heterogeneity explained	Y	Y	Y	Y	Y	N
Publication bias assessed	N	Y	N	N	Y	N
Conflict of interest	Y	Y	Y	Y	Y	Y
Total score	13/16	12/16	13/16	13/16	13/16	6.5/16
Pass/fail	Pass	Pass	Pass	Pass	Pass	Fail

One cross-sectional study was eligible for quality appraisal. This was done using the Appraisal tool for Cross-Sectional Studies (AXIS). As shown in Table 4, the study passed after scoring 14 out of 19 possible points (>70%). Item 14 was not applicable because there were no non-responders. For most items, answering "no" was equal to zero points except for item 13 ("concerns about response rate"), in which case answering "no" was worth 1 point. This is because item 13 being absent is to the benefit of the study, whereas other items being absent is to the detriment of the study.

**Table 4:** Summary of AXIS AXIS: Appraisal tool for Cross-Sectional Studies [16] Y: yes, N: no, NA: not applicable

Checklist	Doumas et al. (2021) [35]
Clear objectives	Y
Appropriate design of study	Y
Justified sample size	Y
Clearly defined target population	Y
Appropriate sample frame	Y
Appropriate selection process	Y
Measures to address non-responders	N
Appropriate risk factor(s) and outcome(s)	Y
Variables measured appropriately	N
Determination of statistical significance	N
Description of methods	Y
Description of basic data	Y
Concerns about response rate	N
Information about non-responders	NA
Internal consistency of results	Y
Presentation of all analyses results	Y
Justification of authors' discussion/conclusion	Y
Limitations discussed	N
Reporting of conflict of interest or funding	Y
Ethical approval attained	N

For the one eligible case report, we employed the Joanna Briggs Institute (JBI) Critical Appraisal Checklist for Case Reports to assess its quality. Each item received 1 point for every "yes" (Y) provided. As shown in Table 5, the case report passed the appraisal with a score of at least 5/7 (>70%). The item on the identification of adverse events is not applicable due to the case report having no reported adverse events.

**Table 5:** Summary of the JBI Critical Appraisal Checklist for Case Reports JBI: Joanna Briggs Institute Critical Appraisal Checklist for Case Reports [17] Y: yes, N: no, UN: unclear, NA: not applicable

Checklist	Luo et al. (2021) [36]
Patient demographics described	Y
Timeline of history presented clearly	Y
Current clinical condition presented clearly	Y
Clear description of investigations	Y
Clear description of interventions	Y
Clear description of post-treatment condition	Y
Adverse events identified and described	NA
Takeaway lesson from case provided	Y
Total score	7/7
Pass/fail	Pass

Six cohort studies were critically appraised using the Newcastle-Ottawa Scale (NOS) Quality Assessment Scale for Cohort Studies. As shown in Table 6, one study passed. We accepted a cohort study if it scored a minimum of 7 points (>70%). Note that "comparability of cases and controls" is worth a maximum of 2 points as per the assessment tool guidelines, while the other items are worth 1 point. The lack of this item, namely, comparison groups, is the chief weakness of the failed studies.

**Table 6:** Summary of the NOS Quality Assessment Scale for Cohort Studies NOS: Newcastle-Ottawa Scale [18] The passing score is 7/9.

First author, year	Items in the checklist									
	Adequate case definition	Representative cases	Control selection	Control defined	Comparability of cases and controls	Exposure ascertainment	Cases and control ascertainment	Rate of non-response	Total score	Pass/fail
Cai et al. (2022) [37]	1	0	1	1	0	1	1	1	6/9	Fail
Fengler et al. (2021) [38]	1	1	1	1	2	1	1	1	9/9	Pass
Juknevičius et al. (2021) [39]	1	0	1	1	1	1	1	0	6/9	Fail
Mahfoud et al. (2022) [40]	1	0	1	1	0	1	1	1	6/9	Fail
Orekhov et al. (2022) [41]	1	0	1	1	0	1	1	1	6/9	Fail
Rosch et al. (2023) [42]	1	0	1	1	0	1	1	1	6/9	Fail

The Revised Tool to Assess Risk of Bias in Randomized Trials (RoB 2) was used in the quality appraisal for the eight eligible RCTs. To be accepted, studies needed to have an overall risk-of-bias judgment of "low risk." For a study to be "low risk," overall, there must not be more than one incidence of "some concerns." As shown in Table 7, four RCTs passed. Allocation concealment refers to blinding of the patients or outcome assessors. Failing to address allocation concealment, and the randomization of said allocation, led to studies failing the appraisal. Another issue was missing outcome data.

**Table 7:** Summary of RoB 2 RoB 2: Revised Tool to Assess Risk of Bias in Randomized Trials [19] LR: low risk, HR: high risk, SC: some concerns Passing criteria: The study must be low risk to qualify.

First author, year	Randomization	Allocation concealment	Missing outcome data	Measurement of outcome	Reported result selection	Overall judgment
Azizi et al. (2023) [43]	LR	LR	LR	LR	LR	LR
Bergland et al. (2021) [44]	SC	SC	SC	SC	LR	HR
Kario et al. (2022) [45]	LR	SC	LR	LR	LR	LR
Kario et al. (2023) [46]	LR	LR	LR	LR	LR	LR
Kirtane et al. (2023) [47]	LR	LR	LR	LR	LR	LR
Mahfoud et al. (2021) [48]	SC	SC	SC	SC	LR	HR
Rader et al. (2022) [49]	LR	SC	SC	LR	LR	SC
Townsend et al. (2024) [50]	SC	SC	SC	LR	LR	HR

Some included studies did not neatly fall into any of the other study classifications, so we used different quality appraisal tools to grade them. We used the Quality Assessment Tool for Before-After (Pre-Post) Studies With No Control Group tool for two studies without a control group. As shown in Table 8, both studies failed to reach the required 70% of total points. Notable weaknesses shared by both studies included the inadequate sample sizes (for comparison, all included RCTs had more patients) and, to exacerbate the sampling issue, a considerable loss to follow-up. Consequently, the power of these studies would have been of concern.

**Table 8:** Summary of the Quality Assessment Tool for Before-After (Pre-Post) Studies With No Control Group [20] Y: yes, N: no, CD: cannot determine, NA: not applicable

Checklist	Wang et al. (2024) [51]	Zeijen et al. (2022) [52]
Was the objective clearly stated?	Y	Y
Were the selection criteria clear?	Y	Y
Was the sample representative?	Y	N
Were all eligible participants enrolled?	CD	CD
Was the sample size adequate?	N	N
Was the intervention clear	Y	Y
Were the outcome measures clear?	Y	Y
Was there blinding of outcome assessors?	N	N
Was there a 20% or less loss to follow-up?	N	N
Were statistical tests done?	Y	Y
Were outcome measures taken multiple times?	N	Y
Did the statistical analysis consider individual-level data?	NA	NA
Total score	6/11	6/11
Pass/fail	Fail	Fail

Two non-randomized studies used the Risk of Bias in Non-randomized Studies - of Interventions (ROBINS-I) assessment tool. These studies required an overall "low risk" grading for acceptance; as shown in Table 9, both studies failed. The studies needed to be "low risk" for all domains to receive an overall "low risk" judgment. There was not enough information given to demonstrate the avoidance of selection bias, for example, the inclusion of blinding and randomization.

**Table 9:** Summary of the ROBINS-I ROBINS-I: Risk of Bias in Non-randomized Studies - of Interventions (ROBINS-I) assessment tool [21] LR: low risk, MR: moderate risk Passing criteria: The study must be low risk to qualify.

Author, year	Confounding	Selection bias	Classification bias	Deviation from interventions	Missing data	Measurement of outcomes	Selection of reporting result	Score
Mahfoud et al. (2021) [53]	LR	MR	LR	LR	LR	LR	MR	MR
Sesa-Ashton et al. (2023) [54]	LR	MR	LR	MR	MR	LR	LR	MR

Discussion

History of Renal Denervation

To understand the odyssey of renal denervation (RDN), it is necessary to provide a brief recounting. At the risk of belaboring, we remind the reader that the sympathetic nervous system is one of the many spokes in the wheel of blood pressure regulation [22]. The earliest attempt at targeting the nervous system to treat hypertension was the splanchnicectomies of the mid-20th century. However, this modality was not tenable due to significant morbidity and the novel choice of more feasible oral anti-hypertensive agents [26]. Animal studies from the 1970s demonstrated this crucial link between the sympathetic nervous system and hypertension [25].

In 2009, the first human trial, SYMPLICITY-HTN 1, was undertaken and displayed significant BP reduction. Notably, there was no control group in this study, and so, building on this trial, SYMPLICITY-HTN 2 was done with a control group but no allocation concealment. SYMPLICITY-HTN 2 (2010) continued the trend of significantly reduced BP. Naturally, this led to enthusiasm for this novel modality. Finally, SYMPLICITY-HTN 3 (2014), a randomized, single-blind, sham-controlled trial, was undertaken with great anticipation. Disappointingly, there were no significant differences in BP reduction between groups 12 months out from the procedure [24].

This sharp reversal in fortunes for RDN led to better, newer trials. These included the SPYRAL and RADIANCE trials. This new generation of trials was better because various measures were taken, such as negating potential confounders, and improving the RDN technique by targeting more renal vessels, and updating the equipment used. In addition, the trials were randomized, sham-controlled, and blinded [26]. The use of blinded, sham-controlled trials was a valuable source of data with greater validity and reduced risk of bias. The results provided renewed evidence for the efficacy of RDN in reducing BP. With a growing pool of literature that reaffirms its efficacy, the promise of RDN has been reinvigorated of late. In fact, the changing attitude toward RDN can be proven in more practical terms such as hypertension guidelines.

Different regions of the world have gradually updated their guidelines to accept the efficacy of RDN as clinical evidence ebbed and flowed. Looking at the evolution of hypertension guidelines worldwide from 2018 to 2022, the pattern is clear; the proof of renal denervation was initially seen as too ambiguous in 2018 to being seen as a valid management option for specific patient populations by 2022. Hypertension guidelines in Europe, Malaysia, and Taiwan all embraced RDN as a valid adjunct or treatment option [27]. One example of RDN's increasing currency was a 2021 cross-sectional study surveying 36 Greek hypertension experts on their views toward the efficacy and safety of renal sympathetic denervation (RDN). The vast majority of participants considered RDN efficacious and safe in reducing BP while acknowledging that the long-term data on both efficacy and safety needs to be increased [35].

Mechanism of Action/Pathophysiology

**Figure 2:**
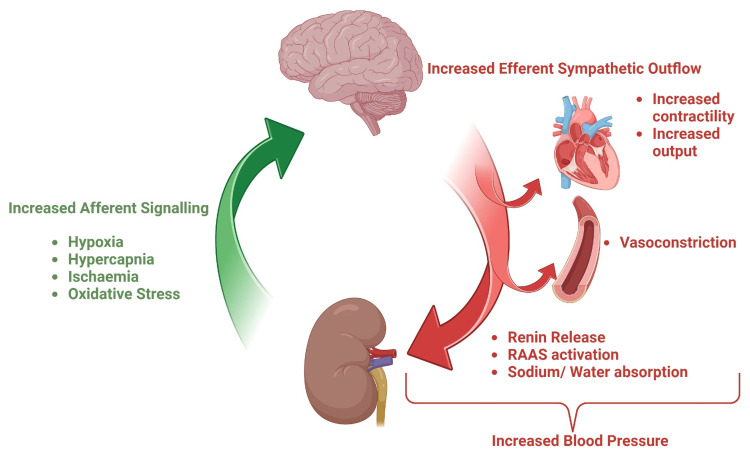
Relationship between the kidneys, the sympathetic nervous system, and blood pressure RAAS: renin-angiotensin-aldosterone system We created the figure using BioRender.com.

As mentioned in the introduction to this review and at the outset of this discussion, the sympathetic nervous system is an important contributor to blood pressure. In recognition of this, there have been pharmacological agents that target this component, such as beta-blockers, and experimentation on the nerves themselves [22,25,26]. The current iteration of this pioneering approach to a ubiquitous disease is renal sympathetic denervation (RDN). RDN involves ablating the renal sympathetic nerves, which traverse along the renal arteries, to disrupt the sympathetic nervous system, which is a key regulator of blood pressure [23]. This relationship is elucidated in Figure 2. Generally speaking, RDN is done via an intra-arterial catheter [24]. A number of methods have been experimented with, and we shall outline them.

To begin, we turn our attention to radiofrequency renal sympathetic denervation. The aim of this method is to interrupt afferent and efferent sympathetic nerve fibers snaking across the surface of the renal arteries while avoiding injury to surrounding tissue. As the name suggests, this ablation is achieved by administering highly focused radiofrequency energy, which is thermal in nature [24]. Multiple administrations of this ablative energy may be applied as the catheter is circumferentially rotated to cover sufficient surface area. Possible complications of such a procedure include local endothelial injury and thrombosis [36]. The very first generation of experiments, the SYMPLICITY trials, utilized radiofrequency ablation delivered via a mono-electrode radiofrequency catheter. The disappointing results of the third SYMPLICITY trial necessitated a better second generation of renal denervation. Enter the Spyral catheter. This newer catheter delivered multielectrode radiofrequency in the SPYRAL trials. The improved technique in the SPYRAL trials included targeting both main renal arteries and branches [26].

**Figure 3:**
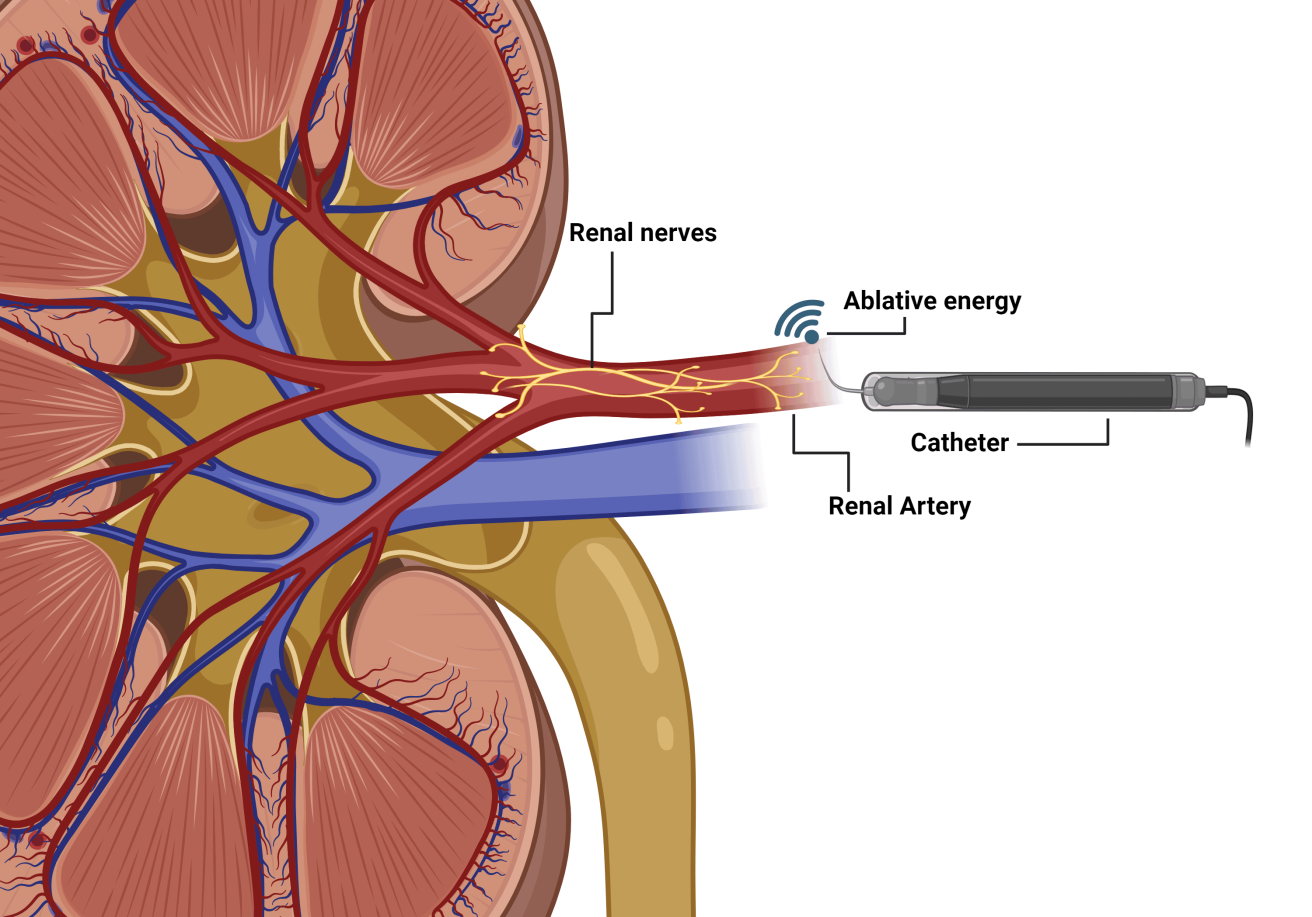
Diagrammatic overview of renal sympathetic denervation Ablative energy is applied circumferentially to renal sympathetic nerves, transmitted through the adventitia of the main renal artery and/or its branches. We created the figure using BioRender.com.

Beyond radiofrequency, renal denervation via ultrasonic waves (uRDN) was also explored, most notably using the PARADISE ultrasound ablation system [24,43]. The principle of this method involves utilizing an intra-arterial balloon catheter that circumferentially applies ultrasonic energy to the sufficient depth required to disrupt the adventitial renal nerves [24]. Before the delivery of the procedure, angiography was performed to identify the target vessels via CT or MRI. Sonification occurred in sequence and targeted the main renal arteries and accessory renal arteries [43]. Local endothelial injury was avoided by a water-cooling mechanism [24]. The general principle behind renal denervation is displayed in Figure 3.

Even more novel methods of renal denervation are being experimented on. In the summary of findings, we mention a case of CT-guided chemical renal denervation being performed in China. In contrast to the modalities above, this case utilized a percutaneous approach. The chemical in question used for this method of nerve ablation was anhydrous ethanol. A CT scan was done to determine the ideal path and angle of the percutaneous path. Afterward, a needle was advanced along this path until it was close enough to the chosen renal artery to ablate the adventitial nerves. The ethanol spreads along the adventitia of the renal artery via diffusion, thereby covering more surface area with fewer needed applications. The authors of that case report offered this as an inherent advantage compared to the radiofrequency method mentioned above [36].

Summary of Main Findings in the Literature

This review contains various study types, but we acknowledge that randomized controlled trials (RCT) offer the most robust evidence. Therefore, we turn to each of these trials to summarize their essential findings.

Ultrasound renal sympathetic denervation: The RADIANCE II trial examined ultrasound renal denervation (uRDN) with affirming results. Two months post-procedure, daytime ambulatory SBP was greatly reduced from baseline with uRDN (mean: -7.9 mm Hg) versus the sham procedure (mean: -1.8 mmHg) (P<0.001) [43]. A pooled analysis of ultrasound renal denervation trials (RADIANCE II, RADIANCE-HTN SOLO, and RADIANCE-HTN TRIO) provides valuable increased power and generalizability of critical findings [47]. At the two-month mark, this pooled analysis of over 500 patients showed that daytime ambulatory systolic blood pressure decreased from baseline by 8.5 mmHg in the ultrasound RDN group to a mean of 141.8 mmHg. For the sham control group, the reduction in the same parameter was 2.9 mmHg to a mean of 147.9 mmHg (P<0.001) in favor of uRDN. BP was reduced from baseline with uRDN, outperforming sham consistently across various BP categories including office SBP (-10.4 mmHg versus -3.4 mmHg) and home SBP (-8.4 mmHg versus -1.4 mmHg) [47]. Given that these pooled results demonstrate a consistent BP reduction with uRDN versus sham confers excellent confidence in the efficacy of uRDN at the very least, if not RDN in general.

However, not all trials on uRDN were very reassuring. In the REQUIRE trial [45], the results of the primary endpoint at three months demonstrated that from baseline, the 24-hour ambulatory systolic blood pressure reduction was of no significant difference between the renal denervation group (-6.6 mmHg) and the sham control group (-6.5 mmHg) (P=0.971). The authors suggested that the study design was at fault and, in a post hoc analysis, reported discrepancies in medication adherence between the treatment and control groups. They noted that among patients who had good medication adherence from the start, uRDN was superior in lowering BP, but in patients with poor adherence at the beginning of the trial, there was a trend for the sham group to do better. This was suggested to be due to the sham group improving their medication adherence as the study went on [45].

Radiofrequency renal sympathetic denervation: In the SPYRAL HTN-ON Med trial [46], BP response to radiofrequency renal denervation using the multielectrode Spyral catheter was investigated. It is worth mentioning that the patients in this trial continued using anti-hypertensives. Thirty-six months out from the procedure, comparing the RDN group to the sham group displayed significantly lower 24-hour SBP, morning and nighttime SBP. Twenty-four-hour SBP was reduced to -20.2 mmHg for RDN versus -10.2 mmHg for sham (P=0.0087). Morning SBP was reduced to -23.9 mmHg for RDN versus -8 mmHg for sham (P=0.029). Nighttime SBP reduction was -20.8 mmHg for RDN versus -7.2 mmHg for sham (P=0.0011) [46]. A side note is the question of whether the new Spyral catheter is the cause of improved outcomes compared to first-generation trials. Past reviews have disagreed on whether the newer multielectrode catheter system outperforms the first-generation mono-electrode catheter in various trials [9,30]. Still, the SPYRAL trial has resurrected the stock of RDN since the SYMPLICITY-HTN 3 results.

Chemical renal sympathetic denervation: We would forgive the reader for thinking renal denervation is only secondary to radiofrequency or ultrasonic energy. However, finally, we turn to a third type of ablation. We present the case study of an 80-year-old hypertensive female who received CT-guided ethanol renal denervation with exciting results [36]. The patient sustained a notable SBP reduction four years after this procedure. At baseline, three days before the procedure, this patient had an SBP of 178 mmHg, which was reduced to 145 mmHg by year 4 post-procedure. In addition, while the patient required three anti-hypertensives before the procedure (without satisfactory control), by year 4 post-procedure, the patient was satisfactorily controlled and completely medication-free [36].

*Morbidity and Mortality*

Efficacious control of hypertension is expected to manifest long-term in reductions of complications linked to uncontrolled hypertension. Efficacy is not merely lowering ambulatory blood pressure 24 hours or 24 months after an RDN procedure, but it also, in theory, should reflect fewer incidences of adverse cardiovascular events. In one cohort study [38], this is demonstrated by the disparity between RDN responders and non-responders. Responders were defined as having ≥5 mmHg reduction in 24-hour SBP between baseline measurements and three months out from RDN. It was found that these responders had a lesser incidence of cardiovascular and ischemic events. Further evidence of a causal relationship was suggested when BP reduction was categorized in 10 mmHg steps. Here, the risk for major adverse cardiovascular events was proportionally decreased relative to the 10 mmHg gradations of 24-hour ambulatory blood pressure measurements (ABPM) [38].

As mentioned before, the main aim of this study was to ascertain the efficacy of RDN. However, it is worth a brief highlight that clinical trials also demonstrated an unfailing safety profile, with low rates of adverse effects due to the RDN procedure [32]. Whenever adverse effects were observed, they were related to vascular access, such as puncture site pain or local hemorrhaging. However, the aforementioned possible thrombosis or vascular stenosis was not reported in the trials included in this review.

Clinical Implications

What are the clinical implications of renal denervation? Hypertension guidelines, which have revolved around the stepwise regimen of medication prescriptions for decades, are now being updated to include this novel option [27]. The emerging data is rapid and dynamic. We are in a transitional phase as it relates to finding a place for renal denervation in the grander hypertension management strategy.

Several general principles may help traverse this new frontier on the hypertension map. To start, guidelines should clearly outline the parameters and methods for diagnosing resistant hypertension, as these patients are the prototypical targeted population for this procedure. We come to a related point: which patients most benefit from renal denervation? In addition to being diagnosed with resistant hypertension, there are potential specific predictors of RDN feasibility. Exemplary attributes predictive of a superior BP response to uRDN, in particular, include a significant baseline BP, higher heart rate, and the presence of orthostatic hypertension [47].

Another potential application is for patients who cannot use or tolerate anti-hypertensive medications, such as the case of renal failure. Patient preference is also essential to the dialogue between physician and patient; many patients want to use fewer medications or none. Intriguingly, RDN seems to possess an inherent advantage over anti-hypertensives; it has an "always-on" effect [45,46]. Why is this an advantage? Pointedly, there is no need for patient adherence or consistency. For a variety of medical issues, it is attractive to develop strategies that rely less on patient adherence or that are less vulnerable to patient error, such as an intrauterine device (IUD) versus an oral contraceptive pill.

Perhaps an easier task is to point out which class of patients are less suitable for RDN. Caution should be taken with patients who have structural renal abnormalities, such as smaller blood vessel diameters. Secondary hypertension due to causes such as renal artery stenosis and hyperaldosteronism is also listed as disqualifying [27].

Future Directions

We recommend that guidelines be clear on their definition of resistant hypertension as they decide which patients are eligible for renal denervation. We have defined resistant hypertension in the introduction of this review but acknowledge that in perusing the literature, there have been differences in the definition [22].

As of 2024, the cumulative body of more recent, better-designed RCTs and systematic reviews post SYMPLICITY-HTN 3 demonstrate the efficacy of RDN in treating resistant hypertension. The next stage in the evolving landscape of RDN is to establish a comprehensive and sustained reduction in the long-term complications of hypertension. These complications include cardiovascular outcomes, renal outcomes, and cerebrovascular outcomes, to name a few, but the cost of uncontrolled hypertension is all-encompassing and steep [24,25].

Concerns have been raised about the potential reinnervation of the renal nerve, as demonstrated in previous animal studies [24,28,31]. Hitherto, this has not been documented in current RDN trials. This would have implications for long-term viability in RDN. However, such concerns lie outside the scope of the current body of literature, and therein lies a potential for future examination.

It has been noted in previous reviews that applying radiofrequency ablation to the main renal artery in addition to the branches of the renal artery had better 24-hour ambulatory BP reduction compared to radiofrequency of the main renal artery only [33]. This finding lends to future considerations of the technical aspects of renal denervation. Suggestions include trials to compare the efficacy of renal denervation to other procedure-based modalities in the treatment of hypertension, such as carotid baroreceptor stimulation and transcranial direct current stimulation [22,23]. Within the scope of renal denervation itself, it would be interesting to systematically explore any differences in efficacy procedural variations of renal denervation, such as ethanol ablation versus radiofrequency ablation.

Limitations

To prove efficacy comprehensively, the long-term complications of hypertension, which can take decades to manifest, need to be demonstrably curtailed. There was a notable paucity of longer-term trials in this review, a feature of RDN's novelty in part. The most extensive follow-up was up to four years, but in truth, renal denervation should be observed well beyond the 10-year mark.

Moreover, we kept our literature search to a four-year span of 2021 to 2024 to analyze more recent developments, which is a small window in the overall literature on renal denervation. Other factors that would diminish the depth of information offered in this study include the exclusion of studies that were not written in English, not free to access, and not found in the five databases searched. The exclusion of non-English studies may point to the importance of multilingual co-authorship papers. This study included 19 studies that passed the critical appraisal, but only four RCTs made it to the final review. Given the aims of this systematic review, there should have been a greater share of RCTs to discuss, thereby providing greater confidence in the results reported. Beyond this, this review's variation of study designs may lead to inconsistent findings due to the potential introduction of bias or prejudice, particularly in studies that are not randomized trials. Finally, we acknowledge that there will be some degree of subjectivity when utilizing specific components of various appraisal tools.

## Conclusions

This review aimed to determine if renal denervation had proven efficacy in reducing blood pressure in patients with resistant hypertension. To summarize, renal denervation (RDN) has significantly lowered systolic blood pressure compared to sham control, and this reduction is maintained for multiple years. This effect is observed whether the renal denervation is applied via radiofrequency, ultrasonically, or chemically. It is especially efficacious in patients who have a higher baseline blood pressure. In addition, the renal denervation procedure demonstrates a sturdy safety profile, and any noted complications were directly related to vascular access. Future considerations should examine the efficacy of one renal denervation modality against another, such as ultrasound RDN against radiofrequency RDN, and compare renal denervation to other procedural strategies such as carotid baroreceptor stimulation.
